# Patient-derived xenograft models of colorectal cancer in pre-clinical research: a systematic review

**DOI:** 10.18632/oncotarget.11184

**Published:** 2016-08-10

**Authors:** Kai M. Brown, Aiqun Xue, Anubhav Mittal, Jaswinder S. Samra, Ross Smith, Thomas J. Hugh

**Affiliations:** ^1^ Northern Clinical School, University of Sydney, Sydney, New South Wales, Australia; ^2^ Upper GI Surgical Unit, Royal North Shore Hospital and North Shore Private Hospital, Sydney, New South Wales, Australia; ^3^ Cancer Surgery and Metabolism Research Group, The Kolling Institute of Medical Research, Royal North Shore Hospital, Sydney, New South Wales, Australia

**Keywords:** patient-derived xenograft, colorectal cancer, systematic review, PDX, animal model

## Abstract

**AIMS:**

We sought to objectively assess the internal and external validity of patient-derived xenograft (PDX) models as a platform in pre-clinical research into colorectal cancer (CRC). Metastatic disease is the most common cause of death from CRC, and despite significant research, the results of current combination chemotherapy and targeted therapies have been underwhelming for most of this patient group. One of the key factors limiting the success of translational CRC research is the biologically inaccurate models in which new therapies are developed.

**METHODS:**

We used the PRISMA (Preferred Reporting Items for Systematic Reviews and Meta-Analyses) checklist and SYRCLE (Systematic Review Centre for Laboratory animal Experimentation) guidelines to search Ovid MEDLINE and Embase databases up to July 2015 to identify studies involving PDX models of CRC where the model had been validated across multiple parameters. Data was extracted including host mouse strain, engraftment rate, site of engraftment, donor tumour source and development of metastases in the model.

**RESULTS:**

Thirteen articles satisfied the inclusion criteria. There was significant heterogeneity amongst the included studies, but overall the median engraftment rate was high (70%) and PDX models faithfully recapitulated the characteristics of their patient tumours on the microscopic, genetic and functional levels.

**CONCLUSIONS:**

PDX models of CRC have a reasonable internal validity and a high external validity. Developments in xenografting technology are broadening the applications of the PDX platform. However, the included studies could be improved by standardising reporting standards and closed following the ARRIVE (Animals in Research: Reporting *In Vivo* Experiments) guidelines.

## INTRODUCTION

Colorectal cancer (CRC) is the third most common cancer and the fourth most common cause of cancer death worldwide [1, 2]. Over 55% of the global burden of disease occurs in developed countries and overall 5-year survival rates are approximately 65%^3^. Metastatic disease is the most common cause of death, and although resection can cure most patients with stage I cancer, 40% of patients with stage II-III cancer will develop metachronous, locoregional or distally recurrent cancer [3, 4]. Furthermore, up to 20% of patients have metastatic disease on presentation, most commonly in the liver [5]. Despite enormous international efforts to discover new therapeutic strategies for CRC, current treatments with combination chemotherapy and targeted monoclonal antibodies have not dramatically changed overall survival rates. Frustratingly, up to 95% of new drugs that eventually get to Phase III trials ultimately are shown to be ineffective in humans [6].

One factor likely to play a role in the failure of phase III trials is the biologically inaccurate pre-clinical models in which many drugs are developed [7-9]. Although there is no widely accepted tool to score the effectiveness of a given biological model [10, 11], it is possible to assess worth based on compliance with the ARRIVE (Animals in Research: Reporting *In Vivo* Experiments) guidelines [12] and the ability to maintain internal and external validity [13]. Internal validity refers to an experiment's ability to identify causal relationships and partly depends on being able to control for confounders. External validity refers to the applicability to the real-world context and depends, among other things, on a model's predictive power.

Cell lines commonly used in basic and translational research are maintained over many passages and frequently have little resemblance, genetically or functionally, to the tumours from which they originated. Cell line-derived animal models lack the complex contribution of the human stromal and immune compartments of the tumour microenvironment (TME) as well as intra-tumoural clonal heterogeneity [14]. There is now good evidence highlighting the importance of these factors for drug resistance [15-18], tumour invasion [19], metastasis [20, 21] and recurrence [22]. More complex animal models of tumour biology exist including carcinogen-induced models, genetically engineered mouse models (GEMMs), as well as patient-derived xenograft (PDX) and humanized mouse models. Each of these has different strengths and limitations for a given research question and this has been reviewed extensively elsewhere [23, 24].

PDX mouse models, whereby tumour from individual patients is grafted into an immune-deficient animal, stand out amongst these advanced platforms as most accurately resembling the human tumour counterpart genetically, and in many respects functionally [25-27]. PDXs have also been shown in numerous studies to maintain intra-tumoural clonal heterogeneity [28-30] and to most accurately reflect drug efficacies in the clinical setting [7, 31]. There are variations in methodology of grafting and of host mouse strain that can influence engraftment rate, metastatic potential, and attrition of stromal and immune components of the TME.

We hypothesize that PDX models of CRC can be assessed for internal and external validity by characterizing the various models, describing the range of translational applications published to date, and by assessing future potential opportunities PDX present for CRC research.

## RESULTS

**Figure 1:**
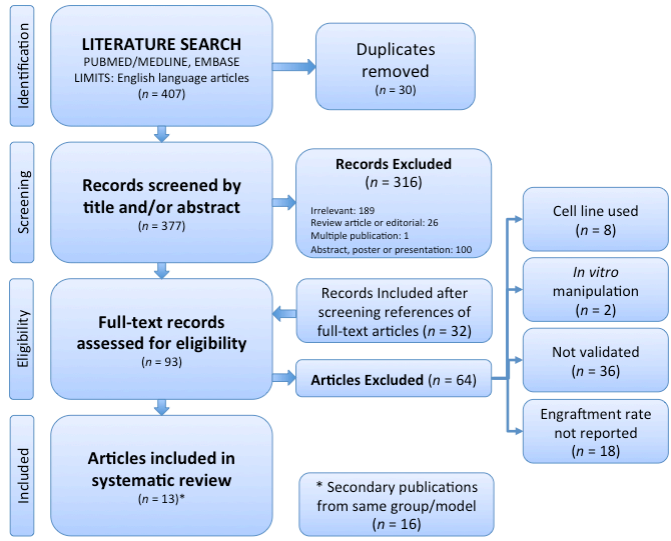
PRISMA Flow Chart

The systematic review process was conducted according to the PRISMA framework [32] (Figure 1) and considering SYRCLE [33] guidelines. From a search of Ovid MEDLINE and Embase databases, 377 unique records were identified. 316 records were excluded after screening the title and/or abstract on the basis of relevance (most commonly due to the use of a cell line-derived xenograft or alternate tumour type), and not fulfilling the requirement of being a primary research article. There was one study published in two different journals. The reference lists of the remaining 61 records were screened to identify a further 32 records, resulting in 93 full-text articles assessed for eligibility. Of these, 64 were excluded, most commonly on the basis that no validation of the PDX was performed. This left 29 articles for qualitative analysis. Amongst these, 13 articles described model validation and these were the subject of the systematic review. The remaining 16 articles referenced some of these 13 studies as a subsequent publication from that research collaboration. The details of the core 13 articles are outlined in Table 1. The other 16 secondary studies were considered only in terms of the application of the model to minimise selection bias.

**Table 1:** Fully validated Patient-Derived Xenograft Models of Colorectal Cancer

First Author	Year	Total number of patient tumours engrafted	Graft Source (engraftment rate)		Overall engraftment Rate	Number of P1 mice grafted per patient	Size of graft (mm^3^ or cell number)	Mouse Strain	Xenograft Type (Heterotopic vs Orthotopic)	Metastases generated in model
			Primary CRC	mCRC tumour^#^						
**Fichtner[61]**	2004	35	33^*^	2^*^	43% (> 3 passages)	6 − 8	25	nu/nu	Heterotopic	
**Dangles-Marie[44]**	2007	26	10 (90%)	16 (69%)	77%	5	Not specified	nu/nu	Heterotopic	
**Mischek[67]**	2009	10	All metastases		60%	3	8 (then disassociated into suspension)	SCID/beige	Heterotopic	
**Linnebacher[46]**	2010	48	All primary		72% overall (71% from cryopreserved)	6 − 8	27	NOD/SCID and nu/nu	Heterotopic	
**Bertotti[47]**	2011	150	All metastases		87%	2	Not specified	NOD/SCID	Heterotopic	
**Jin[48]**	2011	1	Matched primary and metastasis		60% primary 80% liver met	20	12	BALB/c nude	Heterotopic	
**Kim[50]**	2012	20	2 successful^*^	12 successful^*^	70%	5	8-27 (then disassociated into suspension)	NOD/SCID	Heterotopic	
**Julien[34]**	2012	85	58 (60.3%)	27 (70%)	64%	2	50	nude	Heterotopic, 41 orthotopic in parallel	32% of ortho
**Monsma[40]**	2012	18	All primary		50%	Up to 5	≤3mm long axis	nu/nu	Heterotopic	
**Puig[36]**	2013	40	32 (84%)	8 (100%)	88%	Not specified	1 × 10^5^cells (1 × 10^6^for orthotopic)	NOD/SCID	6x Orthotopic	Yes
**Chou[43]**	2013	50	34 (53%)	16 (56%)	58%^**^	Not specified	1 × 10^4^ − 2 × 10^6^cells	NSG	Heterotopic^^^	
**Lee[42]**	2014	10	All primary		100%	Not specified	10	BALB/c nu/nu or NOD/SCID mice	Heterotopic	
**Cho[49]**	2014	143	Mixed primary and metastases, numbers not specified		67%	5 − 6	1 − 2	BALB/c nude	Heterotopic	

# mCRC – metastatic colorectal cancer

* Authors did not stratify engraftment rate according to tumour source

^ 46 specimens subcutaneously grafted into the flank, 14 heterotopically grafted into the subrenal capsule

** 33 of 57 surgical specimens from 27 out of 50 individual patients were engrafted

### Study characteristics

**Figure 2:**
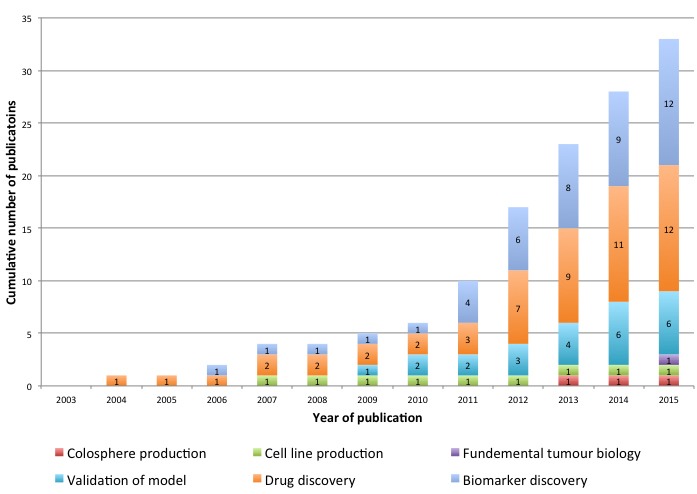
PDX Models of CRC; cumulative number of publications by application and by year of publication

Significant heterogeneity existed between studies making a collated analysis difficult (Table 1). There was a steady increase in the number of publications over time, with half of the studies published since 2012 (Figure 2). The median number of patient tumours successfully engrafted was 27 (range 1 − 97). Studies either engrafted a combination of primary and metastatic specimens (*n* = 8), all primary CRC specimens (*n* = 3) or all metastatic CRC specimens (*n* = 2). Studies validated the animal models across a variety of different passages. Julien et. al. [34] used 10 passages but most studies were within 5 or less passages (data not shown). This is consistent with the PDX literature involving other tumour types showing that xenografts are able to maintain a high level of genetic fidelity for at least 5 passages [7, 35].

### Xenografting methodology

Xenografting methodology was highly variable across the studies as noted in Table 1. Athymic nude (nu/nu) and NOD/SCID (NOD.CB17-*Prkdc^scid^*/J or NOD.CB17-Prkdc^scid^/NcrCrl) mice were equally the most frequently used hosts (*n* = 5), with BALB/c nude (C.Cg/AnNTac-*Foxn1^nu^* NE9) mice being the third most common (*n* = 2). SCID-beige (C.B-17/IcrHsd-Prkcd^scid^Lyst^bg^) and NSG (NOD.Cg-*Prkdc^scid^ Il2rg^tm1Wjl^*/SzJ) were also each used in two separate studies. Almost all the studies exclusively implanted the grafts within a heterotopic subcutaneous or subrenal pocket (*n* = 11). Four studies engrafted a single cell suspension of enzymatically disassociated patient tumour, but only two of these quantified the number of cells engrafted. The remaining studies used whole tumour fragments. Some studies used multiple fragments per mouse, across a wide range of tumour graft sizes (1-50mm^3^), however other studies did not specify. Similarly, a wide range was observed (2-20 mice) for the number of mice engrafted in the first (P1) generation, and some studies did not specify. Only two studies adopted an orthotopic method. Puig et. al. [36] enzymatically digested the tumour specimen into a single cell suspension and then injected a known number of cells into the caecal wall. In contrast, Julien et. al. (2012) sutured a portion of the tumour specimen to the serosa of traumatised murine caecal wall.

### Engraftment rates in PDX of CRC are high

Engraftment rates were described differently across studies with some reporting the relative number of successful human-to-mouse passages while others reported the relative number of successful passages overall. The median overall engraftment rate was 70% (interquartile range 17%). Overall, the rates are significantly higher than PDX models using other primary tumour streams such as breast [37], prostate [38], bladder [39], pancreatic [40] or melanoma [41]. Lee et. al. [42] published an engraftment rate of 100% in both the first and second generation of mice, which is well above that described in the CRC PDX literature. However, this study involved small numbers of patients (*n* = 10) and only relatively advanced tumours were selected for grafting (40% of patients were Stage IV and 60% were Stage III, 80% showed lymphovascular invasion and only 10% were well-differentiated).

PDX models using a NOD/SCID strain had the highest engraftment rate, followed by BALB/c nude and nu/nu, with median rates of 78% (*n* = 4), 67% (*n* = 2) and 63% (*n* = 5), respectively. There was a statistically significant difference in engraftment amongst NOD/SCID versus nu/nu strains (*p* < 0.0001, OR 3.3, 95% CI = 2.08 − 5.33). Of only four studies [34, 36, 43, 44] that reported engraftment rate according to primary versus metastatic tumour graft, there was no statistically significant difference in the individual studies or the pooled data (*p* = 0.37, OR 0.74, 95%CI = 0.39 − 1.4). This contrasts with observations elsewhere in the literature that metastases have an enhanced ability to successfully engraft [41, 45]. Puig et. al. [36] did however, note that metastasis-derived grafts trended towards a shorter (mean ± SD) latency time than primary tumour grafts (46.6 ± 21.7 versus 68 ± 34 days, respectively), and that node-negative or well differentiated primary tumours had a significantly lower engraftment rate compared with more biologically aggressive primary tumours. The preparation of the tumour graft prior to implantation may also be important although no studies specifically examined this. Interestingly, Linnebacher et. al. [46] found no statistically significant difference in engraftment rates between fresh and cryopreserved grafts (74% versus 71%).

### Orthotopic PDX of CRC can develop endogenous distant metastases

The only two CRC PDX models that developed endogenous metastases used an orthotopic method of engraftment (i.e. the tumour was implanted in or near the site of the original source of the tumour rather than in a metastatic site). This is consistent with observations from other tumour types where subcutaneous engraftments almost never produce metastasis [23]. In one of the included studies, 13 of 66 orthotopically engrafted tumours (19.7%) generated distant metastases (to liver and lung) as detected by positron emission topography (PET) and confirmed histologically ex vivo [36]. This excludes peritoneal carcinomatosis that conceivably could be deposited during orthotopic engraftment. Interestingly, the only orthotopically implanted liver metastasis-derived graft did not result in metastases within the model. In another study there was no difference in metastasis rate noted at necropsy (32% overall) in orthotopic PDXs derived from primary versus metastatic tumours [34]. Both of these studies observed the orthotopic xenografts for up to 90 days.

### PDX models of CRC maintain a high degree of microscopic, genetic and functional fidelity to the original donor tumour

In addition to hematoxylin and eosin staining and histological comparison to the original tumour, the included studies validated the various models by examining preservation of key driver gene mutations, most commonly *KRAS, BRAF* and *PIK3CA* (9 studies); gene expression (8 studies); copy number variations (2 studies) and protein expression (predominantly via immunohistochemistry). Three studies looked at preservation of microstatellite instability status. These comparative parameters are outlined in Table 2.

**Table 2:** Validation methods and parameters used to demonstrate PDXs resemble their donor patient tumours

First Author	Year	Validation Method						
		Histology	Driver Gene Mutations	Gene Expression	Copy number Variation	Proteomics	Immunohistochemistry	Other
**Fichtner[61]**	2004	Yes	*KRAS*	ND	ND		EpCAM , CEA, p53, Ki-67, Topoisomerase Ia and IIa	
**Dangles-Marie[44]**	2007	Yes	ND	qRT-PCR	ND			
**Mischek[67]**	2009	Yes	ND	TaqMan low density expression array including Bcl-2, cyclin D1, CDC25B, CDK inhibitor 1B, Erb-b2, K-ras, Met and Myc, EGFR, CTNNB1	ND		CEA, CK8/18, CK20	
**Linnebacher[46]**	2010	Yes	ND	ND	ND			MSI status
**Bertotti[47]**	2011	Yes	*KRAS, NRAS, BRAF, PIK3CA, HER2*	ND	Yes			
**Jin[48]**	2011	Yes	*KRAS, BRAF, EGFR, and PIK3CA*	Affymetrix GeneChip Microarray	ND	Akt, pAkt, ERK, pERK, MAPK, pMAPK, mTOR, mTOR, EGFR, VEGF, Caspase-3, PCNA, GAPDH	CK-20, CDX-2, VEGF, EGFR	
**Kim[50]**	2012	Yes	*TP53, APC; AKT1, BRAF, FBXW7, CTNNB1, KRAS, PIK3CA, EGFR, KDR, FCGR2A, FCGR3A, ERCC1*	Affymetrix GeneChip Microarray	Yes			MSI status
**Julien[34]**	2012	Yes	ND	Affymetrix GeneChip Microarray	ND			
**Monsma[40]**	2012	Yes	*KRAS, PIK3CA*	Affymetrix GeneChip Microarray	ND			
**Puig[36]**	2013	Yes	*BRAF, KRAS, APC, PIK3CA*	Nanostring	ND		b-catenin, caspase-3, chromogrannin A, CK20, EpCAM, Ki67, MUC2, Villin-1	Some correlation of clinicopathalogical characteristics
**Chou[43]**	2013	Yes	*APC*	RNA-seq including PROM1, ALCAM, CD44, CD24	ND		HLA-1, CEA, CK20, Ki-67, CD31, EPCAM, E-Cadherin, PD1, vimentin, fibronectin, CD4, CD8, CD3	
**Lee[42]**	2014	Yes	*TP53, KRAS, PIK3CA, APC, FBXW7, STK11, MET, SMARCB1, ATM, MLH1, PTEN, ERBB2*	ND	ND			
**Cho[49]**	2014	Yes	*BRAF, KRAS, PIK3CA, TP53, APC*	Agilent 60K expression microarray	ND		CK20, CK7, CEA	Short tandem repeat analysis, MSI status

All studies showed a strong preservation of tumour and stromal architecture and histological differentiation. Furthermore, there was evidence that engrafted tumour cells maintained pleuripotency by differentiating into appropriate proportions of mucinosecretory, absorptive and enteroendocrine cells [36], as well as maintained their histopathological subtypes [34]. Protein expression was similar between matched patient tumour and PDX tumour pairs when examined by western blot or immunohistochemistry.

Correlation between key genetic lesions in patient tumours compared to PDX tumours was reported inconsistently. Generally, key genetic lesions were well preserved in PDXs and were noted to occur at similar frequencies to that published in the literature [34]. Puig et al. [36] found 100% concordance amongst 12 matched patient-PDX tumour pairs whereas Lee et. al. [42] found only 80% concordance across 10 pairs, with two PDXs developing a new mutation in both PIK3CA and FBWX7. However, Bertolli et al. [47] noted that wild-type cases persisted unaltered through serial passages and further validated the genomic stability of their PDX model by showing that copy number variation (CNV) between matched pairs was preserved amongst early passages. Similarly, Julien et al. [34] found high genomic stability in CNV for up to 10 passages amongst 90% of cases (34/38 matched pairs). The remaining four matched pairs had very high CNV and thus considered highly genetically unstable, or very low CNV that reduces the accuracy of the CNV assay.

With regard to maintenance of gene expression, correlation between early passage PDXs and original patient tumours was high overall, and in some studies very high with Pearson correlation coefficients (r) ranging between r = 0.86 and r = 0.99 [40, 43, 44, 48]. However, these data are based on limited subsets of successful engraftments and many studies reported these observations differently. Some describe close clustering patterns on gene arrays but methods of measuring clustering are not consistently reported [34, 49, 50]. Puig et. al. [36] noted perfect gene expression clustering of mucinous adenocarcinoma PDX subtypes compared with non-mucinous adenocarcinoma subtypes. In contrast, Dangles-Marie et. al. found that only 2 of 7 paired PDXs and patient tumours had a high gene expression correlation (r = 0.912 and 0.815) [44]. This study used real-time PCR to examine a focused set of 69 genes in one pair and only 17 genes in the remaining 6 pairs, which might explain the different results.

Several studies noted that, of the genes that were different between paired PDXs and patient tumours, many were down regulated and were associated with stroma or the immune system [34, 43, 47]. Furthermore it has been observed that human stroma is often rapidly replaced by murine stroma across the first few passages [51]. Some authors suggest this difference in gene expression is due to the inability of human molecular probes to detect murine stromal analogues and the fact that the animal host is immunodeficient. Consistent with this theory is the observation that there is greater correlation for global gene expression patterns between subsequent PDX generations than there is between patient tumours and the first generation PDX [34, 43].

From a functional standpoint, orthotopic PDX models stood out as being able to generate ‘primary tumours’ and distant lung and liver metastases at similar rates observed in patients [34, 36]. Furthermore, three of the studies specifically examined PDX response rates to cetuximab [34, 47] and systemic chemotherapy [34, 50] and found they reflected clinical response rates. Importantly, they also successfully identified cetuximab-resistant *KRAS* mutant and wild type *KRAS*/mutant *BRAF* or *PI3K* subtypes.

### Validated PDX models of CRC are being used as platforms for multiple applications

Of the 13 included articles in the present review which described a validated PDX model of CRC, a further 16 articles were subsequently published using these models and so these were also considered when evaluating current applications of PDX models in CRC research. Four articles used their model in multiple applications, with 33 total applications across 29 studies. These are illustrated by year of publication in Figure 2.

Applications of PDX models in CRC research ranged across six broad domains, including biomarker discovery [19, 34, 47, 52-60], drug discovery [36, 44, 48, 50, 60-66], PDX model validation alone [40, 42, 43, 46, 49, 67], research pertaining to fundamental tumour biology [19], cell line production [44] and colosphere production [68]. Biomarker discovery and drug discovery (12 studies each) were the most frequent applications, representing 70% of the published studies. The median year of publication was 2012, where there was a particular increase in the number of published studies relating to drug discovery.

### Potential sources of bias

Due to the large variation in methodology and reporting across studies no quantitative analysis of bias could be performed. However, a number of common issues appeared that could serve as potential sources of bias. Overall, there was poor compliance with ARRIVE guidelines with all studies failing to address at least one ARRIVE item [12].

Across most studies, selection bias was likely high. Dangles-Marie et al. [44] only used samples taken from patients with advanced cancers undergoing palliative resections. Only four of 13 included studies gave a comprehensive description of the clinico-pathological characteristics of the patient tumours [34, 36, 42, 47]. All other studies failed to adequately describe how patients were selected for xenografting or their clinical details including whether they had been exposed to prior chemotherapy. Two studies completely changed their methodology during the study by altering the strain of mouse used [42, 69].

Seven of the studies involved a treatment cohort of PDX mice [34, 36, 44, 47, 48, 50, 61]. However, only two [47, 48] reported randomisation of mice to treatment groups although there was not detailed description of the randomisation process. The remaining studies did not describe the treatment allocation process and no study reported power calculations or methods of blinding in reporting results.

Reporting bias was also high in a number of studies, particularly in relation to engraftment rate where it was often unclear if engraftment success referred to tumour growth per mouse engrafted or per patient tumour. Reporting of gene mutation and gene expression analysis was also highly variable, with only one study reporting precisely at which passage the analysis was performed [42], and four studies failing to quantitatively present the results [47, 49, 67, 69]. Only two studies investigated why some tumours failed to engraft. Bertolli et al. [47] noted that these failures tended not to have mutations in *KRAS, NRAS, BRAF* or *PIK3CA*. In contrast, Chou et al. [43] could not identify any clinico-pathological characteristics associated with failure to engraft.

## DISCUSSION

Animal models in the study of cancer have been the cornerstones of pre-clinical research for the last 50 years. However, there are limitations with regard to external validity particularly in relation to the critical role that intra-tumoural heterogeneity and the TME have in cancer growth, metastasis and drug resistance [20, 70]. Not surprisingly there are relatively few translational success stories from bench to bedside. This is also true in colorectal cancer where the mainstay of adjuvant treatment continues to be toxic platinum-based chemotherapy, and where there are limited biomarkers or high impact targeted therapies [71]. This is the first systematic review of PDX models of CRC providing a comprehensive assessment of the PDX platform as a tool for pre-clinical research.

The major criticism of the internal validity of a PDX model is the difficulty in controlling for variables intrinsic to a patient-derived sample such as uncertainty in quantifying the amount of viable tumour being transplanted, intra-tumoural heterogeneity or by some other confounding genetic trait unknown to the investigator at the time of engraftment [27]. This is in addition to other factors such as the various host mouse stains and grafting methodologies used within and across studies, the use of both primary and metastatic samples and the impact of any neo-adjuvant treatment. Whilst this review found that there was a significant difference (p < 0.0001, OR 3.3, 95% CI = 2.08 − 5.33) in engraftment rate amongst NOD/SCID versus nu/nu strains, this result should be considered with a high level of caution, as there was significant heterogeneity in xenografting methodology and variation in reporting of results amongst the included studies. All the above variables could theoretically lead to selection pressure in PDX models resulting in more aggressive tumours being overrepresented in successful PDX engraftments. This would be a source of strong selection bias in the studies reviewed.

While some authors suggest that such non-random selection of tumours or clones that bias engraftment may define a PDX model (by revealing clonal dynamics) rather than limit its usefulness [30], findings from the present review suggest that the risk of selection bias in CRC PDX models may be overstated. Firstly, mice can be reliably engrafted with CRC tumours at rate of 70% or more, with a full representation of histopathological subtypes and microsatellite instability (MSI) status [34, 69]. Secondly, despite the small study from Lee et al. [42], there was no definitive correlation in this review between engraftment rate and stage or grade of the donor tumour (as a surrogate for ‘tumourigenicity’). This contrasts with breast cancer models where triple negative tumours are positively selected during engraftment and passage [37] as well as in non-small cell lung cancer PDX models [45]. Further supporting evidence of the reliability of CRC PDX models is that frequencies of mutant and wild-type driver genes usually mirror those found in the clinical setting, including *KRAS, NRAS, BRAF* and *PIK3CA* [36, 47]. Furthermore, those few studies where a new mutations were detected in PDX tumours but not the original patient samples could reflect low-frequency clones that were below the threshold necessary for detection in the original tumour [42, 47, 49]. This conclusion is consistent with other evidence showing that a library of PDX models are able to display the intra-tumoural heterogeneity of their original patient tumour, both in CRC and breast cancer [28, 29, 72]. If therefore in CRC, the PDX models can be reproducibly engrafted and mimic the spectrum of clinical disease, the effect of selection pressure on internal validity could be regarded as minimal.

External validity of animal models (how well observations in the model translate to clinical practice) relies on adequate representation of the clinical disease. The present review found that PDX models of CRC maintain a high level of genomic, transcriptional and phenotypic fidelity to the original patient tumour. Preservation of mutations in key driver genes between matched patient and PDX tumours ranged between 80-100%. Furthermore, genetic stability was further shown by maintenance of CNV amongst at least 90% of cases for up to 10 passages. Others have observed the pattern of chromosomal instability in CRC PDXs to be maintained over 14 passages [29]. In this review, all but one study (which only examined a small number of genes) found that gene expression patterns were well preserved amongst paired patient and xenograft tumours. Whilst there is no clear ‘cuff-off’, given the varying methodologies and passages used to assess genetic fidelity in this review, as well as the varied degree of stability across passages in other tumour streams [7], the fidelity of CRC PDXs in late passages must be accepted with caution. Nonetheless by the same measure, the breadth of methodologies used clearly demonstrate there is lack of genetic drift across early-mid passages, which further corroborates the internal validity of PDX models in CRC research. Overall, the conservation of histological subtypes, MSI status, key driver mutations and gene expression from patient tumours to their corresponding PDXs demonstrate that, at least in CRC, PDX models faithfully recapitulate a full spectrum of clinical disease.

In several studies, the differences in gene expression that were observed between matched patient and PDX tumours were found to correspond to down-regulated human stromal and immune-related genes. This is not unexpected as it has been shown that co-engrafted human tumour stroma is usually replaced by murine stroma in the immunodeficient host by the third passage [19, 73]. Nonetheless, at the microscopic level, all included studies demonstrated that the histology of the original tumour was maintained. It is unclear as to what degree differences in receptor-ligand homology as co-engrafted TME is replaced by murine stroma may affect the external validity of PDX models in this regard. Having said that, a recent study used this feature of PDX models to show that an engrafted CRC induced a gene expression signature in murine fibroblasts that correlated with the original patient cancer-associated fibroblasts (CAFs), as well as independently predicted clinical outcomes [19]. Clearly, the immune-deficient status of the murine hosts required to accept human tumour samples without rejection is a major drawback in investigating the role of the immune system in tumourigenesis. This issue will need to be addressed in future PDX research.

Despite the drawback of variable stromal preservation and absence of a competent immune system, repeatedly PDX models in CRC have demonstrated excellent predictive power, which can be considered a measure of external validity. Of the included studies that examined PDX response rates to conventional therapies, there was a close correlation with those found in the clinical setting [34, 47, 50]. Furthermore, Julien et al. [34] found a positive correlation between poor xenograft response to cetuximab and *KRAS, BRAF* or *PIK3CA* mutational status, which is also used to predict patient response. Kim et al. [50] were able to show a statistically significant correlation between an oxaliplatin response gene expression signature in a panel of CRC PDX models and an independent clinical cohort. Importantly, all these studies used clinically relevant doses in the PDX. Finally, the observation that orthotopic CRC PDX models generate ‘primary tumours’ and distant lung and liver metastases at similar rates observed to that in patients [34, 36] may be in part due to the fact that orthotopic models more closely resemble the native TME and hence may improve external validity. This advantage is partly offset by the difficultly in accurately measuring orthotopically engrafted tumours, even with sophisticated imaging systems. By contrast, subcutaneous sites of engraftment are straightforward to measure tumour growth, but almost never generate metastases. Together these studies show the applicability of well-designed PDX platforms to the clinical context of CRC.

This review found an increasing number of publications and an expanding range of applications for PDX models in CRC in the published literature. Even within the strict inclusion criteria of this systematic review, publications using PDX models of CRC have doubled in the last three years (Figure 2). The majority of these studies pertained to either drug or biomarker discovery, with cetuximab the most frequently investigated drug, amongst other targeted agents. Using PDX models as an intermediary to generating CRC cell-lines or colospheres is not common despite this technique having far higher success rates than generating primary cell cultures directly from tumours. Interestingly, there has also been interest in using the PDX model to address fundamental CRC biology research questions. One study used a NOD/SCID PDX model to show that a poor prognosis stem/serrated/mesenchymal CRC subtype was characterised by a CAF specific gene signature, challenging the paradigm that mesenchymal and stemness traits are attributed to epithelial tumour cells [19].

There were several limitations of the present review due to the high degree of heterogeneity between included studies. A number of different engraftment methods, mouse strains, experimental endpoints and reporting standards were used which precluded the ability to perform a meta-analysis or funnel plot analysis of bias. None of the studies reported results according to ARRIVE guidelines and all the studies were at high risk of bias. This is an observation that is, unfortunately endemic amongst pre-clinical animal research [74, 75].

## FUTURE DIRECTIONS

As demand for improved translational outcomes in CRC research increases the use of advanced PDX models has expanded as demonstrated in this review. Accordingly, there is a need to ratify uniform reporting standards, such as the ARRIVE guidelines, in order to make pre-clinical animal studies more transparent and suitable for more powerful meta-analyses. This would help ensure that pre-clinical animal studies are upheld to the same standards as their clinical counterparts.

The complexity of *in vivo* models is rapidly advancing which may overcome some of the current limitations of PDXs. Newer mouse strains such as the NSG and NOG (NOD/Shi-Prkdc^scid^ Il2rc^tm1Sug^/Jic) strains have improved engraftment rates compared with other animals which may reduce selection bias [76]. Furthermore, using a so-called ‘omental’ site of engraftment for both non-small cell lung cancer and ovarian cancer in an NSG host has been shown to maintain and expand functional co-engrafted CAFs and tumour-infiltrating lymphocytes [77, 78]. Such strategies of better recapitulating the TME in animal models should hopefully increase their external validity.

The next tipping point in PDX technology will undoubtedly be widespread access to humanised PDX models (huPDX) which have been outlined elsewhere [76]. In short, huPDX create either genetic or cellular chimaeras that combine the advantages of a human-derived tumour with a variably intact immune system. Such a CRC huPDX would be especially relevant due to high intra-tumoural heterogeneity in colorectal cancer, variable immunogenicity (such as microsatellite stable vs. unstable) and other clinical evidence of importance of immune response [79]. Currently available huPDX models are prohibitively difficult to establish or encounter issues with HLA-mismatch and tumour rejection or graft-versus host disease. At present, work is underway to establish transgenic NSG mice that express human HLA-I/II and lack mouse MHC-I/II or express human growth factors and cytokines [24] which may facilitate ‘off the shelf’ huPDX. It therefore is no surprise that PDX ‘banks’ are being established in both America and across Europe, asserting the future of PDX models in basic science and pre-clinical CRC research.

## MATERIALS AND METHODS

The study protocol for this systematic review followed the PRISMA (Preferred Reporting Items for Systematic Reviews and Meta-Analyses) checklist [32] and used the SYRCLE (Systematic Review Centre for Laboratory animal Experimentation) guidelines for relevant additions [33].

### Search strategy

A search of the Ovid MEDLINE and Embase databases was performed up to July 2015 to identify studies involving PDX models of CRC. The search was limited to English language papers. Both medical subject heading and free text searching were used to increase the sensitivity of the search. The search terms included in the Ovid MEDLINE string and the Embase string are shown in Supplementary Appendix S1 and Supplementary Appendix S2, respectively.

Papers were first screened on title and abstract for relevance and eligibility before the full text of remaining papers were screened for eligibility. Additional papers were identified by manually screening the references of any included study.

### Inclusion criteria

Included papers had to be English language primary research articles that used a primary or metastatic colorectal PDX model without any intervening *in vitro* manipulation. Any method of engraftment could be included, however papers were not included if xenografts had undergone significant *in vitro* manipulation (such as cell culture) prior to engraftment. Papers were only included if the engraftment rate was explicitly stated, as the absence of this information might contribute to selection bias in the model or data. Successful engraftment was broadly considered as any xenograft of sufficient volume for downstream ex vivo or *in vivo* study. Lastly, the models described must have undergone a process of validation in addition to comparative haematoxylin and eosin histology in order to sufficiently explore genetic and or phenotypic differences between patient tumour and their corresponding xenografts. Reviews and editorial articles were excluded from this review.

### Data extraction

Each included paper was manually searched for parameters including host mouse strain, engraftment rate, site of engraftment (heterotopic or orthotopic), development of metastases in the model and primary or metastatic donor tumour source. Validation methods including preservation of histology, driver gene mutations, gene expression, copy number variation, immunohistochemistry, proteomics and others were annotated. Where a study did not directly validate the model in the manuscript but instead referenced a previous published validation study, these were grouped accordingly.

### Statistical analysis

Statistical analysis was performed by the 2-tailed χ2 test using Microsoft Excel for Mac 2011 (version 14.5.9) and Prism 6 for Mac OSX (version 6.0f). For all tests, the level of statistical significance was set at *P* < 0.05.

## SUPPLEMENTARY MATERIAL


