# Supplementary material for: Patient-derived xenograft models of colorectal cancer in pre-clinical research: a systematic review
Source: Oncotarget. 2016 Aug 10;7(40):66212–25. doi: 10.18632/oncotarget.11184 (PMC5323228; doi:10.18632/oncotarget.11184)
Supplement: Supplementary file 1 [file oncotarget-07-66212-s001.pdf]

Patient-derived xenograft models of colorectal cancer in pre-clinical research: a systematic review

Supplementary Material

S1 – Ovid MEDLINE Search string

(exp Colorectal Neoplasms/ OR colon* adenocarcinoma*.mp. OR colon* cancer*.mp. OR colon* tumour*.mp. OR colon* carcinoma*.mp. OR rectal adenocarcinoma*.mp. OR rectal cancer*.mp. OR rectal tumour*.mp. OR rectal carcinoma*.mp.) AND (exp Heterografts/ OR exp Xenograft Model Antitumor Assays/ OR xenograft*.mp. AND patient derived.mp. OR explant.mp.)

S2 – Embase Search String

[colorectal AND ('cancer'/exp OR cancer) OR colon* AND cancer* OR colon* AND tumour* OR colon* AND carcinoma* OR colon* AND adenocarcinoma* OR colorectal AND ('tumor'/exp OR tumor) OR colorectal AND ('carcinoma'/exp OR carcinoma) OR 'rectal carcinoma'/exp OR 'rectal carcinoma' OR 'colorectal carcinoma'/exp OR 'colorectal carcinoma' OR 'rectal cancer'/exp OR 'rectal cancer' OR 'colon cancer'/exp OR 'colon cancer' OR 'colon adenocarcinoma'/exp OR 'colon adenocarcinoma' OR 'colorectal cancer'/exp OR 'colorectal cancer' OR 'colon tumor'/exp OR 'colon tumor' OR 'colon carcinoma'/exp OR 'colon carcinoma' OR 'colorectal tumour'/exp OR 'colorectal tumour' OR 'rectal tumor'/exp OR 'rectal tumor'] AND ['xenograft' AND (explant OR 'patient derived')] AND [english]/lim AND [embase]/lim
